# A MoS_2_ Nanoflakes-Based LC Wireless Passive Humidity Sensor

**DOI:** 10.3390/s18124466

**Published:** 2018-12-17

**Authors:** Shujing Su, Wen Lv, Tong Zhang, Qiulin Tan, Wendong Zhang, Jijun Xiong

**Affiliations:** 1 Key Laboratory of Instrumentation Science & Dynamic Measurement, Ministry of Education, North University of China, Tai Yuan 030051, China; sushujing@nuc.edu.cn (S.S.); 18335162736@163.com (W.L.); zhangtongzztt@163.com (T.Z.); wdzhang@sxedu.gov.cn (W.Z.); xiongjijun@nuc.edu.cn (J.X.); 2 Science and Technology on Electronic Test and Measurement Laboratory, North University of China, Tai Yuan 030051, China

**Keywords:** humidity sensor, LC, wireless passive, MoS_2_ nanoflakes, sealed environment

## Abstract

In this study, an LC wireless passive humidity sensor based on MoS_2_ nanoflakes was proposed. The LC wireless passive humidity sensor was optimized by performing HFSS simulations and fabricated via a screen-printing technique. The MoS_2_ nanoflakes were characterized by laser scanning confocal microcopy, scanning electron microscope, and X-ray diffraction. The measurements show the sensor can operate stably for a long time with a hysteresis of 4% RH (relative humidity) in 10–95% RH. At low humidity environment (10–60% RH), the sensitivity of the as-prepared humidity sensor is 2.79 kHz/% RH, and a sensitivity of 76.04 kHz/% RH was realized in a high humidity environment (60–95% RH). In this regard, the sensing mechanism was discussed in the scope of proton transfer theory. The test results also indicate that the response time and recovery time of the prepared sensor are 10 s, 15 s, respectively and between 15~40 °C the sensitivity of sensor was not temperature-dependent in the range of 10~80% RH. In addition, the sensor shows less sensitivity to temperature in the 15–25 °C range at 90% RH. All of these experimental results show that the prepared LC wireless passive humidity sensor can stably monitor the rapidly changing humidity in a sealed and narrow environment for a long time.

## 1. Introduction

Humidity sensors have been widely used in industrial, agricultural, medical, food package and household life [1,2,3,4,5]. A qualified humidity sensor needs to meet the following requirements, including linear response, fast sensitivity, wide humidity range, fast response, low cost, and small hysteresis [6,7]. There have been tremendous efforts towards building humidity sensors employing different sensing mechanisms such as resistive [8], Surface Acoustic Wave (SAW) [9,10], optical fiber [11,12], capacitive [13], and LC [14] technologies, etc. Resistive sensors are inexpensive and exhibit good performance over a medium humidity range, but resistive meters respond weakly at lower humidity. In addition, they have issues of size, yield, current drift and integration with Complementary Metal Oxide Semiconductor (CMOS) circuits [15], which limit the development of resistive humidity sensors. As SAW devices, its operating frequency tends to be affected by the poor signal to noise performance and the required circuit is normally complex and costly [16]. Despite the immunity to electromagnetic interference, optical sensors were limited in many applications due to the expensive and large-scale photoelectric equipment required for support [17]. In contrast, the capacitance and LC resonant type sensors, which operate with capacitance changes, are widely adopted owing to their merits, including good linearity, high sensitivity, less complex signal processing circuits and low power consumption [18]. Compared to capacitive humidity sensors, LC resonance as a non-contact method can remotely measure parameters of interest, making it ideal for monitoring humidity parameters in environments where wired connections are not possible such as deep mines with combustible gases, sealed vessels and so forth, thus LC resonant sensors have obvious advantages in sealed and hazardous environments. Compared to traditional active humidity sensors, they are inexpensive to manufacture, simple in construction and theoretically long in life, making them ideal for both sealed and non-contact measurement environments. Salmerón et al. reported a wireless humidity sensor, but for environments with only 20 to 80% RH, but the measurement range is small and the sensitivity of the sensor is very low [19]. Zhang et al. proposed a LC graphene wireless humidity sensor and it can be used in 15–95% RH realizing a sensitivity of 18.75 kHz/% RH [20]. Wang et al. reported a humidity label for humidity detection in the range 10–90% RH, and the sensitivity is only 1.1 kHz/% RH [21]. Table 1 gives a comparison of other previous works. The sensing performance features of some of the sensors mentioned above are not desirable and need to be improved.

Owing to the high surface area to volume ratio, 2D materials such as graphene, MoS_2_ have been widely studied and demonstrated their gas sensing capability [27,28]. However, some positive properties such as zero band gap, and high hysteresis make graphene limited in many applications [29]. On the other hand, MoS_2_ being as a gas sensing material is gaining more attention due to the unique properties including tunable ban gap, lower intrinsic background carrier density, etc. Moreover, owing to the special molecular structure, there exist many dangling bonds on the edge of the MoS_2_ structure and these provide a large amount active sites interacting with gas molecules, which implies MoS_2_ is a promising material for humidity sensing [30]. Nevertheless, due to the influence of temperature drift on the accuracy of the sensor, there are few studies on sensor humidity monitoring in a variable temperature environment. To our best knowledge, LC wireless passive humidity sensors based on MoS_2_ have not been reported.

In this work, we studied a LC wireless passive humidity based on MoS_2_ nanoflakes. With the help of High Frequency Structure Simulator (HFSS) software, the structure and distance–S_11_ curve of the as-designed humidity sensor was optimized, and the sensor circuit was fixed to the alumina substrate by screen printing technology which is simple to operate and low cost. By dint of laser scanning confocal microcopy (LSCM) we investigated the morphology and topography of the as–sprayed MoS_2_ film and MoS_2_ nanoflakes were characterized by a scanning electron microscope (SEM) and X-ray diffraction (XRD). Then the experimental and simulation results were compared and the prepared sensor was tested over a wide humidity range at room temperature. Moreover, other sensing performances such as hysteresis, the response and recovery time of as-prepared humidity sensor were investigated.

## 2. Materials and Methods

### 2.1. Design and Operating Principle

**Figure 1:**
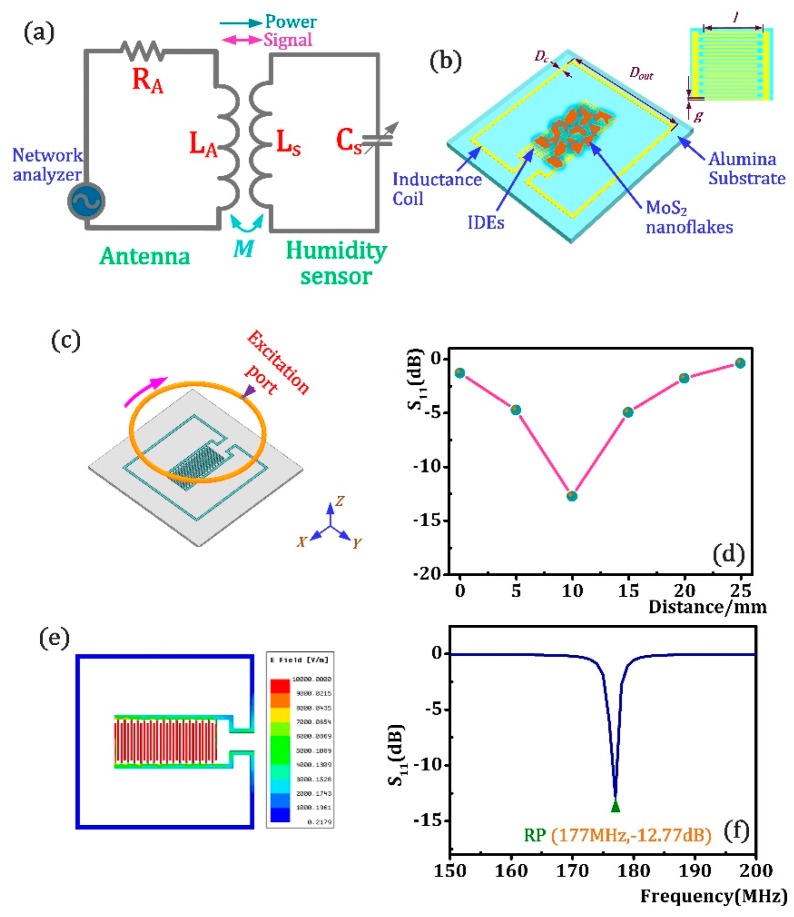
(**a**) Illustrative model schematic of LC wireless measurement; (**b**) as-designed humidity sensor; (**c**) the HFSS simulation model of as-designed humidity sensor; (**d**) the distance-S_11_ curve of humidity sensor; (**e**) electric field distribution of sensor at 10 mm; (**f**) S_11_ versus frequency curve of as-designed humidity sensor based on HFSS simulation at 10 mm.

The principle circuit of the designed LC wireless passive sensor is shown in Figure 1a, in which the external interrogation antenna inductance and the humidity sensor are inductively coupled. The sensor circuit, which consist of stable *L_s_*, and variable *C_s_*, resonates based on Equation (1):

(1) $$C_{f}=\frac{1}{2\pi \sqrt{L_{s}C_{s}}}$$

Therefore, *f*, which is the resonant frequency (RP) of the sensor resonant circuit, is uniquely determined by the variable C. The structure of the proposed humidity sensor is shown as Figure 1b. In this design, a piece of alumina (length × width × thickness: 32 mm × 32 mm × 0.5 mm) was designated as the gauge substrate, on which the external inductor coil and the humidity-sensing capacitance were structured on it. The interdigitated electrodes (IDEs) were chosen as the humidity-sensing capacitance and they are completely sprayed by MoS_2_ nanoflakes as humidity sensing film. Here, the capacitance of the MoS_2_-covered interdigital electrode is calculated using Equation (2) as follows:

(2) $$C_{s}=\frac{Nlh\varepsilon }{g}+\frac{Nl\varepsilon }{2}$$

Where *N* is the number of interdigitated electrodes, *l, g, h* are the length, width, thickness of a interdigitated electrodes, and *ε*, which changes as the MoS_2_ nanoflakes absorbs or desorbs the moisture in ambient environment, is the relative dielectric permittivity of the MoS_2_-moisture mixture. *D_c_* and *D_out_* are the width and the length of inductance. As the capacitance the humidity sensor circuit resonates, the sweep source connecting with an antenna such as network analyzer with a certain frequency range can detect the resonance frequency of humidity sensor circuit, thereby realizing wireless measurement of humidity parameter.

Then, the proposed humidity sensor structure was optimized by High Frequency Structure Simulator (HFSS) software. Figure 1c is the sensor simulation structure model in the analysis, on which the round antenna was vertically coupled with the humidity sensor and excited by a sweep signal ranging from 150–200 MHz. Moreover, the sensor dimensions parameters for simulation analysis are presented as Table 2. In this work, silver was selected as the conductor material for the IDEs and coil. The dielectric constant, dielectric loss and mass density of the ceramic substrate were set to be 9.8, 38 × 10^−3^ and 3960 kg/m^2^, while the bulk conductivity and mass density of the Ag conductor was 6.1 × 10^7^ Siemens/m and 10,500 kg/m^2^. The dielectric constant of MoS_2_ was set to be 12.33. In the analysis, we primarily probed the S_11_ values of sensor in the coupling distance range of 0–30 mm with the increment of 5 mm, and the derived results shown in Figure 1d demonstrated the transmitting limit of the proposed humidity sensor was about 25 mm and the S_11_ value decreases as distance increases in 0–10 mm, and then dramatically creeps with increasing distances in 10–25 mm. The S_11_ value at the distance of 10 mm was higher than at any other distances and its value is up to −12.77 dB which indicates a robust signal level at this position. In this regard, we assume the preferable coupling distance between humidity sensor and antenna was 10 mm owing to the high S_11_ value. Further, the electric field distribution of the sensor at the distance of 10 mm is shown in Figure 1e. The results show that the electric field is mainly concentrated on the interdigital capacitance, while the electric field generated by the peripheral inductance is very weak and can be negligible. The S_11_-f resonance curve of as-designed humidity sensor at 10 mm is shown in Figure 1f, in which, the resonance frequency of sensor is 177 MHz, and S_11_ value is up to −12.77 dB.

### 2.2. Sensor Fabrication

**Figure 2:**
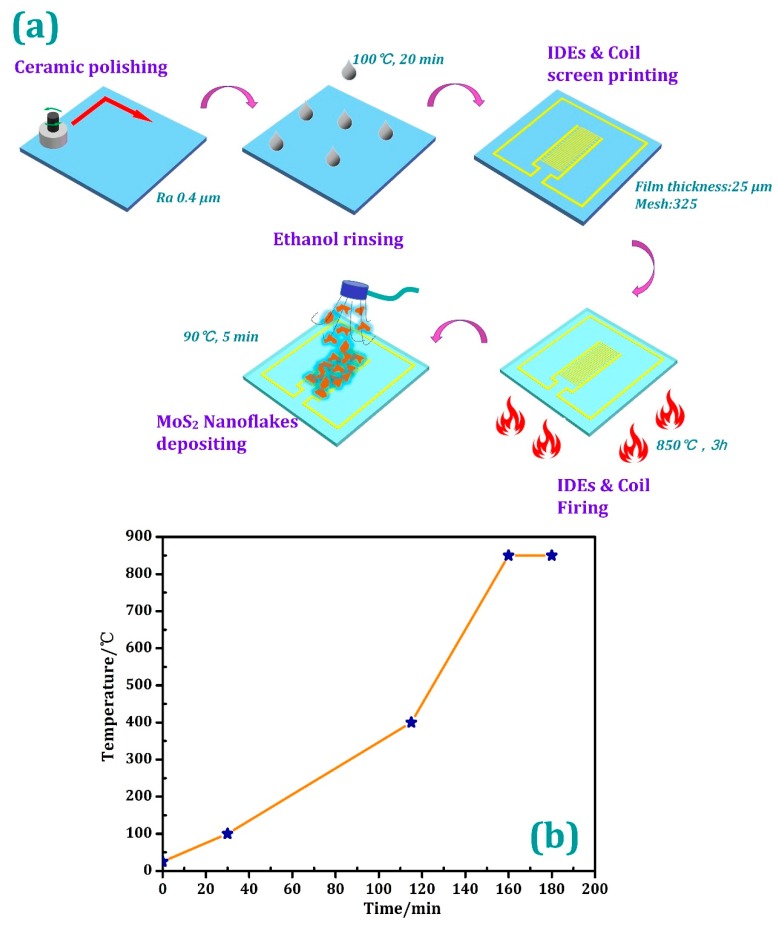
(**a**) The fabrication process and (**b**) the firing curve of the as-prepared LC humidity sensor.

In this work, we fabricated the proposed MoS_2_ humidity sensor using screen printing technology which has the advantages of simple operation, high flexibility, and low cost, etc. when compared with other microelectronic processing methods. CN33-398 Ag conductor (Ferro, Cleveland, OH, USA) with a lower resistance was selected as the inductor and the interdigitated electrodes series material. MoS_2_ nanoflakes (particle diameter: 1.5 μm) were supplied by XFNANO Company, (Nanjing, China). First, to remove the impurities in the MoS_2_ nanoflakes. MoS_2_ nanoflakes (2.0 g) were dispersed in 200 mL deionized water and then magnetically stirred for 10 min, and then the MoS_2_ nanoflakes were collected by vacuum filtration. After repeating the above process three times, the as-prepared MoS_2_ nanoflakes were dried at 90 °C and later dissolved with 200 mL of ethanol for further use. As Figure 2a indicates, the roughness on the top surface of the alumina substrate was polished to 0.4 μm, which facilitated the adhesion between the metal and the ceramic chip. Thereafter, the IDEs and inductance coil were screen-printed on the top of alumina ceramic substrate with Ag conductor after the substrate was rinsed with ethanol and dried at 100 °C for 20 min for hardening the liquid paste. The mesh number and film thickness of the screen were 325 and 25 μm, respectively. Eventually, the screen-printed humidity sensor was fired in a muffle furnace at 850 °C for 3 h to remove the conductor solvent and establish a stable bond between the substrate and the metal. Figure 2b shows the firing curve in the firing process. After the sensor was naturally cooled to room temperature, the as-prepared MoS_2_ nanoflakes solution was evenly sprayed onto the surface of IDEs at room temperature as the humidity sensitive layer of the sensor. After that, the as-sprayed MoS_2_ nanoflakes were dried at 90 °C for 5 min to evaporate the solvent.

**Figure 3:**
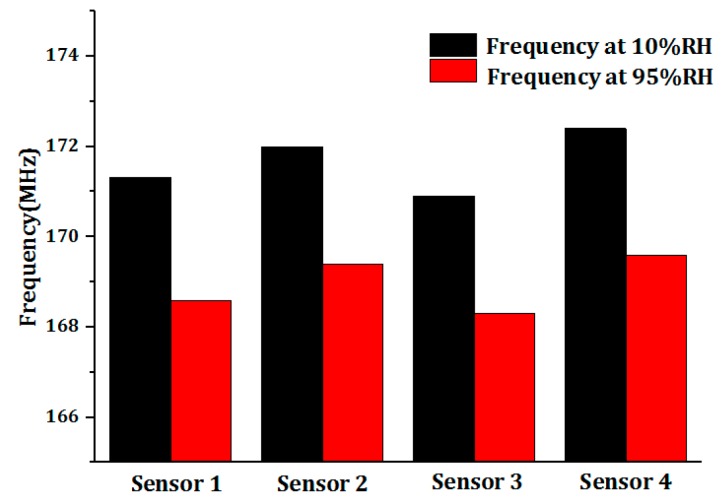
The frequencies of sensors at 10% RH and 95% RH.

**Figure 4:**
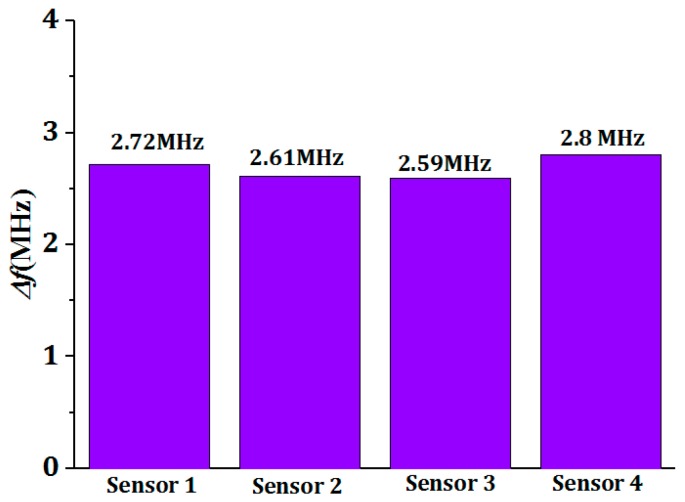
The frequency shift of sensors between 10% RH–95% RH.

Under the same conditions, we fabricated four sensors and the frequency test results of these four sensors at 10% RH and 95% RH of room temperature are presented in Figure 3, which indicate the frequencies of sensors at 10% RH and 95% RH are in between 170.9–172.4 MHz and 168.31–169.6 MHz. At 10% RH, we observed that the frequency difference of sensors tested at 10% RH was tiny and can be ignored. Further, the frequency shift of every sensor in 10–95% RH presented in Figure 4 shows the frequency shift of every sensor was distributed between 2.59 MHz–2.8 MHz. In this regard, we think the humidity sensors we fabricated have good uniformity and can be reproduced.

## 3. Results and Discussion

### 3.1. Characterization of the As-Prepared MoS2 Nanoflakes

**Figure 5:**
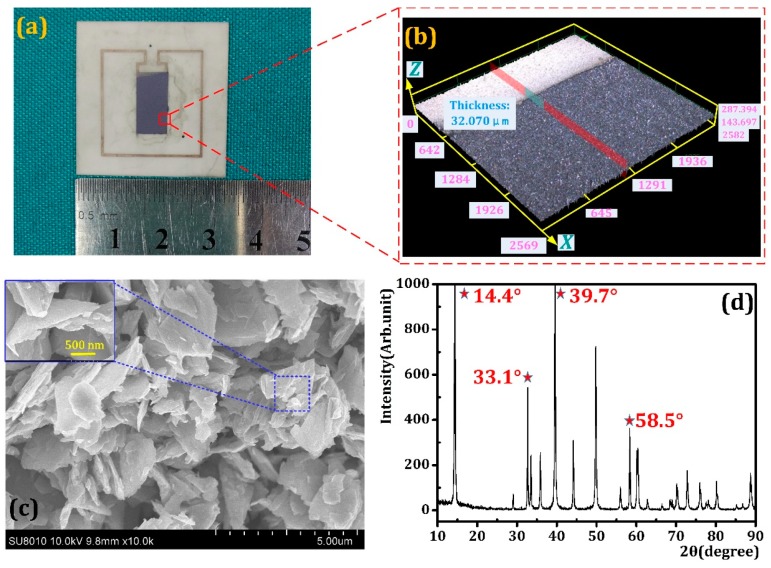
(**a**) The optical image of as-prepared LC humidity sensor; (**b**) the morphology result of as-sprayed MoS_2_ film; (**c**) the microscopy of MoS_2_ nanoflakes; and (**d**) X-ray diffraction patterns of MoS_2_ nanoflakes.

Figure 5a is the schematic of the as-prepared MoS_2_ humidity sensor. By dint of laser scanning confocal microcopy (LSCM) we investigated the morphology and topography of the as-sprayed MoS_2_ film and Figure 5b shows the results. It can be concluded that MoS_2_ nanoflakes were sprayed evenly on the IDEs and the thickness of the MoS_2_ thin film was about 32.07 μm. Further, the MoS_2_ nanoflakes were characterized by the SEM, and the results are shown in Figure 5c. The photograph shows that the particle size of the MoS_2_ nanoflakes is uniform and it has a large surface area to volume ratio. Moreover, owing to the random sequence of nanoflakes, large amount cracks can be absorbed in the SEM image, which may be beneficial for humidity capture. Figure 5d shows an XRD pattern of a MoS_2_ sample prepared by the hydrothermal method. The diffraction peaks of MoS_2_ nanosheets are observed at 2θ of 14.4°, 33.1°, 39.7° and 58.5°, which were attributed with the MoS_2_ nanocrystals of (002), (100), (103), and (110) plane. Moreover, the high and sharp diffraction peak (002) of the as-prepared MoS_2_ samples indicates the sample has good crystallinity and a layered structure.

### 3.2. Sensing Performance of As-Prepared Humidity Sensor

**Figure 6:**
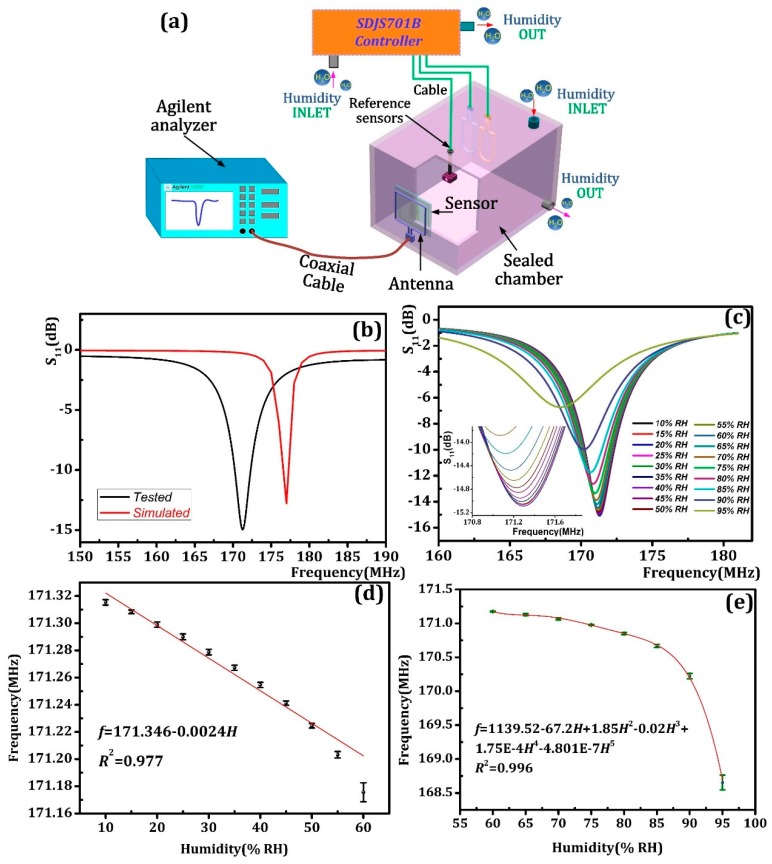
(**a**) the schematic diagram of measurement; (**b**) S_11_ versus Frequency curve comparison of Experimental measurement and HFSS simulation at 10% RH; (**c**) S_11_-f curve of as-prepared humidity sensor in 10–95% RH; (**d**) f-Humidity curve of as-prepared humidity sensor under low humidity conditions (10–60% RH); (**e**) f-Humidity curve of as-prepared humidity sensor under high humidity conditions (60–95% RH).

The sensing performance tests of as-fabricated sensor were arranged in the experimental equipment shown in Figure 6a. The SDJS701B controller was used to adjust the temperature and humidity in the test chamber. Inside the test chamber, the as-prepared sensor was coupled in parallel with the copper antenna and the vertical distance between them is approximately 10 mm. The excitation antenna and an E5061B network analyzer (Agilent, Santa Clara, CA, USA) are connected by an external coaxial cable. The network analyzer processes the frequency and S_11_ data of the humidity sensor in the experimental chamber. According to the simulation results, the frequency sweeping range was set between 150–200 MHz. Note all the tests were processed at room temperature.

First, the as-prepared sensor was placed in the test chamber and the S_11_-f curve of the humidity sensor under 10% RH was extracted as shown in Figure 6b. By comparing the simulation result, it can be found that the resonance frequency of the experimental sensor is 171.25 MHz and has a difference of 6.75 MHz with the simulation result of 177 MHz, indicating that the experimental results are reasonable, and the prepared sensor is almost in line with expectations. We assume the main factor that caused the frequency difference can be that the simulation environment was set as vacuum conditions, which differs from the environment in the test chamber. Moreover, the frequency difference may also be partly due to the process accuracy, which would bring dimension indeterminacy in the printed capacitance and inductance. Further, at room temperature, we investigated the S_11_-f of the as-prepared humidity sensor in a fluctuating humidity environment ranging from 10% RH to 95% RH in increments of 5% RH every 5 min and the S_11_-f curve of the as-prepared sensor is given as Figure 6c, which shows that the signal magnitude known as S_11_ and frequency decrease as humidity variess. Moreover, as the S_11_ value remained below −6.7 dB all the time, this indicates that the humidity sensor shows a robust signal level from 10–95% RH. Further, by repeating the test procedures in 10–95% RH five times, we obtained the f-humidity curve with error bars as Figure 6d and Figure 6f show. It can be concluded from the figure that under low humidity (10–60% RH), the as-prepared humidity sensor frequency decreases linearly with increasing humidity, and the sensitivity is about 2.79 kHz/% RH. In the high humidity range of 60~95% RH, the sensitivity is 76.04 KHz/% RH which is about 27.25 times higher than in the low humidity range.

**Figure 7:**
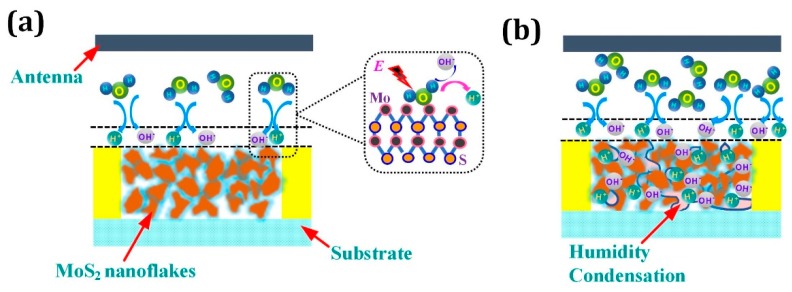
Sensing mechanism (**a**) under low humidity conditions (10–60% RH) and (**b**) under high humidity conditions (60–95% RH).

As Figure 7a depicts, in the low humidity range (10–60% RH), due to the high surface to volume ratio of MoS_2_ nanoflakes and the large amount of cracks induced by the random sequence of nanoflakes, water molecules in the ambient can be absorbed by MoS_2_ nanoflakes. Because of the active defects caused by the Mo atoms on the edge of nanoflakes, the absorbed water molecules were then captured by the defects in the form of Mo-O bonding and ionized by the electric field E between IDEs and dissociated into protons (H^+^) and hydroxyl ions (OH^−^) accumulating and mainly distributing on the surface [31]. Then, the protons were forced and transferred by the electric field, which give rise to the dielectric constant of MoS_2_ nanoflakes. Due to the low humidity level, the water molecule adsorption on the surface was discontinuous and thus the protons transfer was confined and only happened in the discontinuous water clusters of the surface. Consequently, the dielectric constant shift weakly and the capacitance of IDEs was perturbed in a small range, which account for the low sensitivity in 10–60% RH. At high humidity range (60–95% RH), As shown in Figure 7b, the absorbed water molecules aggregated on the surface and the water molecules permeated into the internal space of MoS2 nanoflakes till the water began to condense into large clusters full of water molecules. Thus, a large of water molecules were bonded with the edge defects of MoS_2_ and further dissociated into a large amount of protons (H^+^) and hydroxyl ions (OH^−^). In this way, proton transfer occurred in the bulk volume of the MoS_2_ nanoflakes and became continuous in the condensed water. As a result, the dielectric constant shifts acutely and then the sensitivity was significantly improved compared with the sensitivity in a lower humidity range. Also, as the hydrated water dielectric is 2.2 in the low humidity range, the hydrated water dielectric in the high humidity range is 78, which also gave more capacitance improvement of the humidity sensor in the high humidity range (60–95% RH) [32,33].

**Figure 8:**
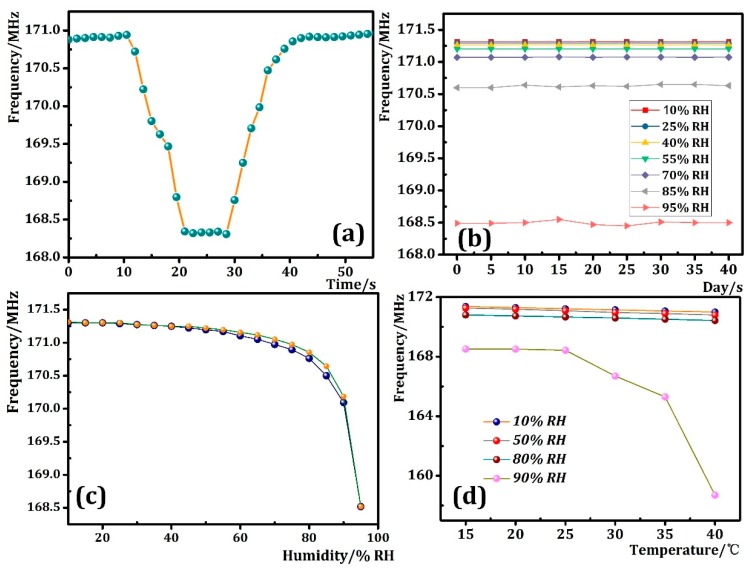
(**a**) The response and recovery, (**b**) long-term stability and (**c**) hysteresis results of as-prepared humidity sensor; (**d**) frequency-temperature curve at different humidity.

Later, we investigated the response and recovery time of the prepared sensor, and frequency-time curve obtained by the experiment is shown in Figure 8a. As can be seen from the figure, the response time and recovery time of the prepared humidity sensor at 10–95% RH are about 10 s and 15 s. This result indicates that the prepared sensor has a faster response and recovery time, and can be used in an environment where the humidity changes rapidly. Next, stability tests of the as-prepared humidity sensor arranged in the test chamber at 10–95% RH were carried out. The frequencies of the humidity sensor were recorded every 5 days for 40 days. As shown in Figure 8b, no acute frequency fluctuation was detected in the stability experiments, which confirms the MoS_2_ nanoflakes are chemically stable to water molecules and the as-prepared humidity sensor has good stability. Also, the hysteresis results shown in Figure 8c suggest that the as-prepared humidity sensor has a maximum hysteresis of about 4% RH, which means it can be used in accurate humidity detection. In addition, we simply tested the effect of temperature on the sensing performance of the prepared humidity sensor under 10% RH, 50% RH, 80% RH, 90% RH. At every humidity step, the temperature was changed from 15 °C to 40 °C and the results are given in Figure 8d. It can be seen from the figure that when the humidity is 10% RH, 50% RH, 80% RH, the linear slope of the sensor frequency is about −0.01491, which means that, the temperature change from 15 to 40 °C has a negligible effect on the sensor frequency in 10–80% RH. By contrast, at 90% RH, the slope of the temperature-dependant curve within the 15–25 °C range is the same as 10% RH, 50% RH, 80% RH. However, when the temperature exceeds 25 °C, the slope of the curve becomes significantly larger. Thus, it can be concluded that the temperature has little effect to the sensitivity of humidity sensor in 15–40 °C below 80% RH and thus it can be ignored.

## 4. Conclusions

This paper presented a MoS_2_ nanoflake wireless passive humidity sensor. The sensor coil is bonded to the alumina ceramic substrate by screen printing technology, which is easy to perform and facile. The sensor structure was optimized by HFSS software simulation and experimental tests show that the prepared LC wireless passive humidity sensor can operate stably at a humidity range of 10~95% RH and has the advantages of high sensitivity and small hysteresis. Under low and high humidity, the sensitivities are respectively 2.79 kHz/% RH and 76.04 kHz/% RH and the response and recovery time of as-prepared sensor is about 10 s and 15 s. In addition, the hysteresis measurements show the sensor has a hysteresis value of 4% RH. Further, it was found that in the 15–40 °C below 80% RH, the temperature has little effect on the sensitivity of the humidity sensor. In summary, the experimental results show that the proposed LC wireless passive humidity sensor can monitor the rapidly changing humidity in a tight, sealed environment.

## Figures and Tables

**Table 1:** The performance comparison analysis of our work and some previous work.

Type	Range (% RH)	Response (s)	Recovery (s)	Sensitivity	Hysteresis	Sensing Material	Reference
**Optical**	20~95	10	60	25.2 mV/% RH		Ag–ZnO	[22]
**Optical**	0~50	50	50~80	78.4 pm/% RH (maximum)	17% RH	SU–8	[23]
**SAW**	10~90	10	20	60 kHz/% RH (maximum)		polyaniline & polyvinyl alcohol	[24]
**Microwave**	5~95	32	25	0~1		SnO_2_ nanoparticles	[25]
**RFID**	15–95			3.7 kHz/% RH		Polyimide	[26]

**Table 2:** Dimension parameters of MoS2 humidity sensor.

l	g	w	IDEs Couples	Dc	Dout	Coil turn
5	0.2	0.04	32	0.5	25.5	1
